# The association between coronary artery disease and osteoporosis: a population-based longitudinal study in Taiwan

**DOI:** 10.1007/s11657-022-01128-3

**Published:** 2022-07-08

**Authors:** De-Kai Syu, Shu-Hua Hsu, Ping-Chun Yeh, Yu-Feng Kuo, Yen-Chun Huang, Ching-Chuan Jiang, Mingchih Chen

**Affiliations:** 1 Department of Orthopedics, Fu Jen Catholic University Hospital, Fu Jen Catholic University, No. 69, Guizi Rd., Taishan Dist., New Taipei City, 24352 Taiwan, Republic of China; 2 Department of Family Medicine, Fu Jen Catholic University Hospital, Fu Jen Catholic University, No. 69, Guizi Rd., Taishan Dist., New Taipei City, 24352 Taiwan Republic of China; 3 Graduate Institute of Business Administration, College of Management, Fu Jen Catholic University, No. 510, Zhongzheng Rd., Xinzhuang Dist., New Taipei City, 242062 Taiwan Republic of China; 4 Artificial Intelligence Development Center, Fu Jen Catholic University, No. 510, Zhongzheng Rd., Xinzhuang Dist., New Taipei City, 242062 Taiwan Republic of China

**Keywords:** Coronary artery disease, Osteoporosis, National Health Insurance Research Database, Taiwan

## Abstract

**Purpose:**

This large population-based study is the first to analyze the association between coronary artery disease (CAD) and osteoporosis (OP) from the National Health Insurance Research Database (NHIRD) in Taiwan to determine if CAD is associated with OP.

**Methods:**

Data from NHIRD, a national, population-based, retrospective, matched cohort study of 23 million patients, were collected to recruit two matched cohorts: with (*n* = 192,367) and without (*n* = 192,367) CAD. The Cox model was used to compare the incidence rate ratio and crude hazard ratio (HR) between the two cohorts for osteoporotic fracture and OP.

**Results:**

The CAD cohort had a significantly increased risk for vertebral compression fracture, with an adjusted HR of 1.74 (95% CI, 1.60–1.89). The cumulative incidence of OP was also statistically higher in the cohort versus without CAD (11.6% vs. 5.6%; *p* ≤ 0.0001, log-rank) during the 10-year follow-up period. The Cox model showed a 2.04-fold increase in the incidence of OP in the CAD cohort, with an adjusted HR of 2.04 (95% confidence interval [CI], 1.99–2.08).

**Conclusions:**

A positive association exists between CAD and development of subsequent osteoporotic fracture and OP. Patients with CAD have a significantly increased risk of developing vertebral compression fracture and a higher incident rate ratio of OP.

**Trial registration:**

IRB approval number: No. C108094 on February 19, 2020.

## Introduction

Coronary artery disease (CAD) and osteoporosis (OP) are major causes of mortality and morbidity in the older adult population [1–3]. The prevalence of both diseases is expected to rise due to increased life expectancies [4–7]. By 2050, the world’s population age 60 years and older is predicted to increase almost double from 13 to 25%, according to the World Health Organization [8]. Because OP may progress without symptoms and is associated with age-related fractures, it is now recognized as a major threat, and Taiwan policymaking to address the prevalence of OP reached a critical point in 2005 [9]. According to the Taiwan Ministry of Health and Welfare in 2015, the prevalence of OP was 17.4% in 2001 and 25% in 2011 [9], and CAD ranked the second highest cause of death among women in Taiwan [10]. Both diseases can independently progress to more severe conditions, which will result in a large socioeconomic burden [11, 12].

Previously, CAD and OP were viewed as unrelated and independent of each other, until recent years when epidemiological and biological studies formed concepts and evidence of an association [13–15]. For CAD, a process called *atherosclerosis*, which describes the buildup of fats, cholesterol, and other substances within and outside the artery wall, is influenced by several risk factors, such as obesity, smoking, hypertension, and diabetes, mainly affecting women after menopause [3]. The pathophysiologic mechanism for bone loss in OP is similar in that the process may be accelerated by age. Both diseases involve an endothelium-lined lumen from the artery walls and the osteon from the cortical bone; therefore, it is possible that endothelial dysfunction may be a common precursor to OP and CAD. Previous studies also showed that noninflammatory and inflammatory factors such as elevated C-reactive protein, tumor necrosis factor-alpha (TNF-α), and interleukin (IL)-6 levels have aggravated both atherogenesis and bone resorption [16]. Another possible link seen between CAD and OP is the high concentrations of nitric oxide, which may play a role in bone metabolism through osteoblastic activity, spreading osteoclasts, and bone resorption.

A retrospective study analyzing asymptomatic postmenopausal women in Korea reported that high coronary artery calcium (CAC) scores and obstructive CAD were associated with OP [17]. A community-based study in China concluded that CAD was independently and significantly associated with OP [3]. However, the most recent meta-analysis of cross-sectional studies in 2020 supported that low bone mineral density (BMD) was not associated with the prevalence of CAD. With an increase in the correlation supporting a possible link between the two comparisons that could only be explained by age-accelerated alone, studies have suggested that no relationship exists between OP and CAD. Although advanced science has improved the understanding of pathophysiologic factors and associations shared by both diseases, these two diseases have yet to be sufficiently investigated with regard to the etiologic role of CAD associated with OP. Therefore, the purpose of this study was to examine if CAD is associated with osteoporotic fracture and OP. We hypothesized that CAD correlates with an increased incidence of OP and osteoporotic fracture.

## Methods

### Data source and ethical considerations

Datasets were retrieved from the National Health Insurance Research Database (NHIRD) in Taiwan, which represents > 99% of the Taiwanese population (approximately 23 million individuals), if not all [18]. By providing abundant information for inpatients and outpatients, including detailed demographic information; initiation date for health care; total medical expenses from ambulatory, prescription, and medication services; and more, the NHIRD has often been used by many researchers for hundreds of published studies [19]. The NHIRD diagnosis codes are the *International Classification of Diseases*, Ninth Revision; Clinical Modification (ICD-9-CM); and the Tenth Revision (ICD-10-CM) in Taiwan which was fully adopted from January 1, 2016. Following the NHIRD ethical guidelines, personal patient information was anonymized before accessing; therefore, the Research Ethics Committee waived the requirements for informed consent. This study was approved by the Institutional Review Board and Ethics Committee of Fu Jen Catholic University (IRB approval number: No. C108094).

### Study population

**Fig. 1:**
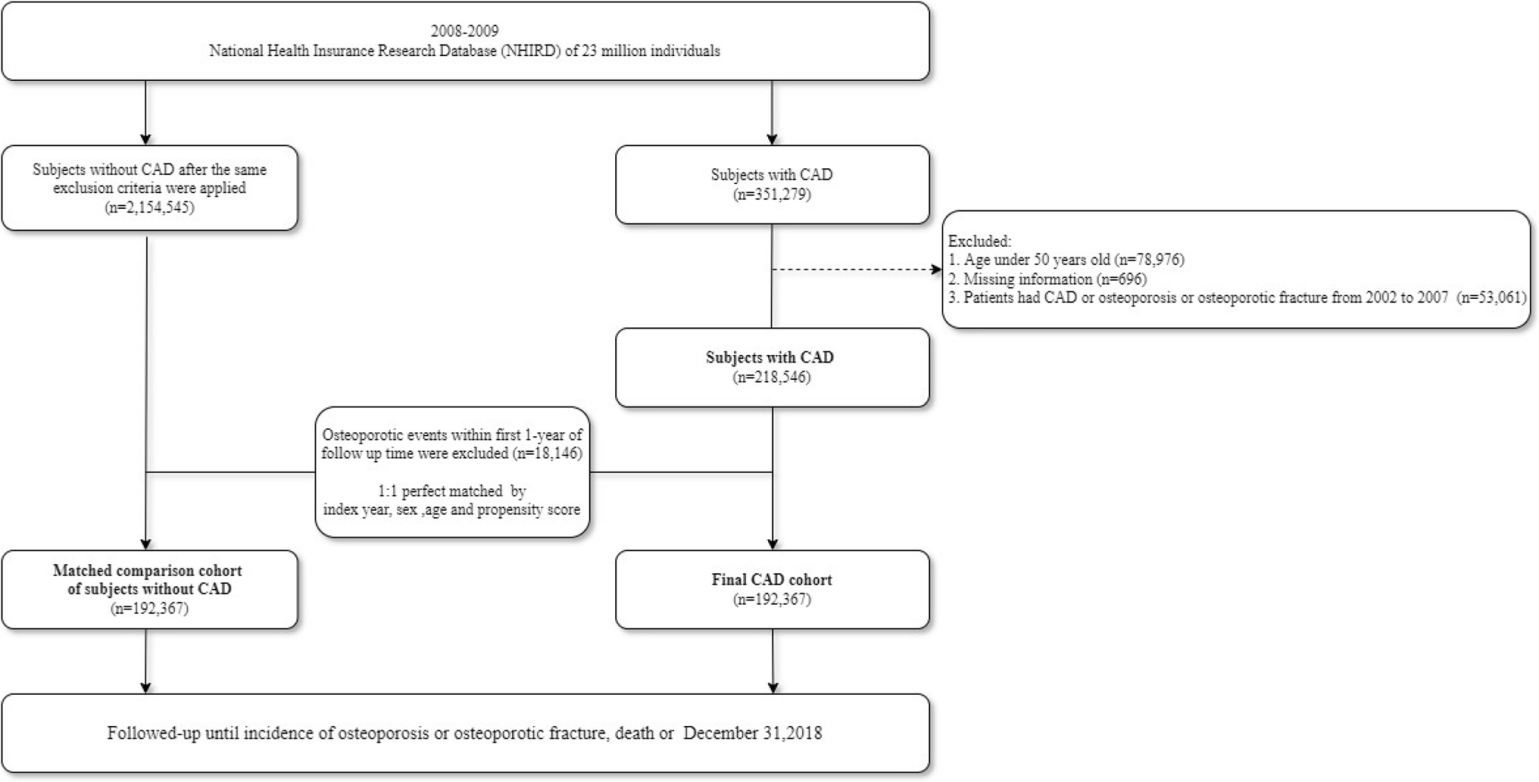
Consort diagram showing detailed steps for assembling the two study cohorts

The study design is a retrospective, population-based, database cohort study. Inclusion criteria were age older than 50 years with physician-diagnosed CAD (ICD-9-CM Code 410 to 414) from January 1, 2008, to December 31, 2009. A total of 192,367 CAD cases were identified after excluding patients meeting any of these criteria: age younger than 50 years (*n* = 78,976); any missing data (*n* = 696); and CAD or OP or osteoporotic fracture (including hip fracture and vertebral compression fracture) from 2002 to 2007 (*n* = 53,061) (Fig. 1). For the comparison group, 192,367 patients without CAD were matched after applying the same exclusion criteria used for the CAD cohort. To minimize any allocation bias, the non-CAD cohort was verified to maintain CAD-free status in the dataset throughout the entire follow-up period. Additionally, the first CAD date was defined as the index date. Patients who were diagnosed with OP after the index date within 1 year were excluded from the study to avoid reverse causation bias. To reduce sampling bias, this study used a 1:1 ratio for propensity score matching with index year, age, and sex. After propensity score matching, the final CAD cohort contained 192,367 patients and the comparison cohort contained the same number of patients. All participants in both cohorts were followed up until the occurrence of OP or OP fracture, death, or December 31, 2018, whichever occurred first.

### Outcome measure and confounding variables

The primary outcome measure was the incidence of thoracolumbar vertebral compression fracture. The secondary outcome measure was physician-diagnosed OP, defined as at least three different medical claims issued in the outpatient setting or at least one claim in the inpatient setting. To avoid the reverse causation phenomenon (protopathic bias), patients with OP outcomes during the first year of follow-up were excluded from the overall risk calculation. In Taiwan, the gold standard for diagnosis of OP is BMD measurement in the spine and hip with a value of 2.5 or more standard deviation (SD) below the young adult female reference mean (*T* score − 2.5 SD or less), based on a white female normative database as the *T* score reference.

Table 1 shows that the two cohorts were balanced for index date, age, and sex after matching. We used categorized insurance premium as a proxy for the participants’ socioeconomic status. Comorbidity data were collected for hypertension, hyperlipidemia, chronic obstructive pulmonary disease (COPD), diabetes mellitus (DM), liver disease, peripheral vascular disease (PVD), congestive heart failure (CHF), stroke, rheumatoid arthritis (RA), and morbid obesity. The Charlson Comorbidity Index (CCI) was added as shown in Table 1.

**Table 1:** Demographics and comorbidities at baseline between the CAD cohort and the age-, sex-, and index date-matched comparison cohort without CAD

	Case (*N* = 192,367)		Compare (*N* = 192,367)		*p*
	*n*	%	*n*	%	
Year					
2008	97,267	50.56	97,564	50.72	0.3382
2009	95,100	49.44	94,803	49.28	
Sex					
Female	87,140	45.29	87,139	45.29	1
Male	105,227	54.71	105,228	54.71	
Age					
50–59	80,787	42	80,787	42	1
60–69	57,304	29.79	57,304	29.79	
70–79	39,173	20.36	39,173	20.36	
80–89	15,103	7.85	15,103	7.85	
Baseline comorbidity					
Hypertension	14,700	7.64	4633	2.41	< 0.001
Hyperlipidemia	48,616	25.27	16,353	8.5	< 0.001
COPD	35,698	18.56	14,669	7.63	< 0.001
Diabetes mellitus	36,928	19.2	14,898	7.74	< 0.001
PVD	10,668	5.55	3839	2.03	< 0.001
CHF	23,026	11.97	5477	2.85	< 0.001
Stroke	25,245	13.12	10,529	5.47	< 0.001
RA	142	0.07	63	0.03	< 0.001
Morbid obesity	106	0.06	28	0.01	< 0.001
Heart failure	6354	3.3	1557	0.81	< 0.001
Liver cirrhosis	2667	1.39	1938	1.01	< 0.001
CCI					
0	110,464	57.42	159,349	82.84	< 0.001
1	25,320	13.16	9318	4.84	
2	21,669	11.26	8439	4.39	
3	14,693	7.64	5920	3.08	
4	9041	4.7	3863	2.01	
5	5051	2.63	2220	1.15	
6 +	6129	3.19	3258	1.69	
Mean (SD)	1.1384 (1.7817)		0.4963 (0.4963)		< 0.001
Mortality	40,089	20.84	46,934	24.4	< 0.001

*SD* standard deviation, *CAD* coronary artery disease, *COPD* chronic lung disease, *PVD* peripheral vascular disease, *CHF* congestive heart failure, *RA* rheumatic arthritis, *CCI* Charlson Comorbidity Index

### Statistical analysis

The basic demographic characteristics were defined as categorical variables, presented as *N* (%), and continuous variables, presented as mean ± SD. Sex and age between the case and the control groups were compared using the chi-squared and *t* tests. Multivariate adjusted Cox proportional hazard regression was calculated for the adjusted hazard ratios (HRs) and 95% confidence intervals (CIs) to estimate the incidences for the case and control groups. Kaplan–Meier curve analyses were conducted to evaluate the cumulative incidences of OP; a log-rank test was used to determine the significance between cases and controls. For all analyses, a two-tailed *p* value of < 0.05 was considered statistically significant. All statistical analyses were conducted using SAS version 9.4 (SAS Institute, Cary, NC, USA).

## Results

Table 1 shows the baseline characteristics of the study participants. A total of 192,367 patients were assessed, with a mean age of 64.26 years. Several significant differences between cohorts were found for the comorbidities of hypertension, hyperlipidemia, COPD, DM, liver disease, PVD, CHF, stroke, and morbid obesity; RA was the only comorbidity without a significant difference (*p* = 0.4234). The mean CCI showed a significant difference between patients with CAD and controls (*p* < 0.001).

For the primary outcome of thoracolumbar vertebral compression fracture, the CAD cohort had a higher cumulative incidence rate than the non-CAD cohort (0.9% vs.0.47%; *p* < 0.001) (Table 2). The secondary outcome of physician-diagnosed OP was present for 22,309 patients with CAD and 10,771controls (cumulative incidence rate 11.6% vs. 5.6%; *p* < 0.001) during the follow-up period (2009–2018).

**Table 2:** Incidence of thoracolumbar vertebral compression fracture, osteoporosis, and the crude and adjusted hazard ratios (HRs) derived from the Cox model for the CAD cohort compared with that from the comparison non-CAD cohort stratified by patient characteristics

	Case (*N* = 192,367)			Compare (*N* = 192,367)		
	*N*	%	Incidence rate	*N*	%	Incidence rate
Compression fracture	1740	0.9	100.8	913	0.47	53.4
Osteoporosis	22,309	11.6	1420.4	10,771	5.6	657.4
Case vs compare						
	Crude hazard ratio (95% CI)		*p* value	Adjusted HR (95% CI)		*p* value
Compression fracture	1.91 (1.76–2.06)		< 0.001	1.74 (1.60–1.89)		< 0.001
Osteoporosis	2.13 (2.09–2.18)		< 0.001	2.04 (1.99–2.08)		< 0.001

Rate incidence per 100,000 PYs

*PYs* person-years

For the primary outcome measure, the CAD cohort had a significant increase in the risk for thoracolumbar vertebral compression fracture, with a crude HR and adjusted HR of 1.91 (95% CI, 1.76–2.06) and 1.74 (95% CI, 1.60–1.89), respectively (Table 2). At 10-year follow-up, 22,309 patients with CAD and 10,771 controls received consistent diagnoses of OP given by a physician. The incidence rates of OP per 100,000 patients per year were 1420 in the CAD cohort and 657 in the comparison cohort, with an incidence rate ratio of 2.16 (Table 2). The crude HR was also statistically significant, showing a 113% increase in the risk with an HR of 2.13 (95% CI, 2.08–2.18). This significant increase of the risk was sustained even after the multivariate Cox model adjusting for the aforementioned confounding factors with an adjusted HR of 2.04 (95% CI, 1.99–2.08; *p* < 0.001) (Table 2).

**Fig. 2:**
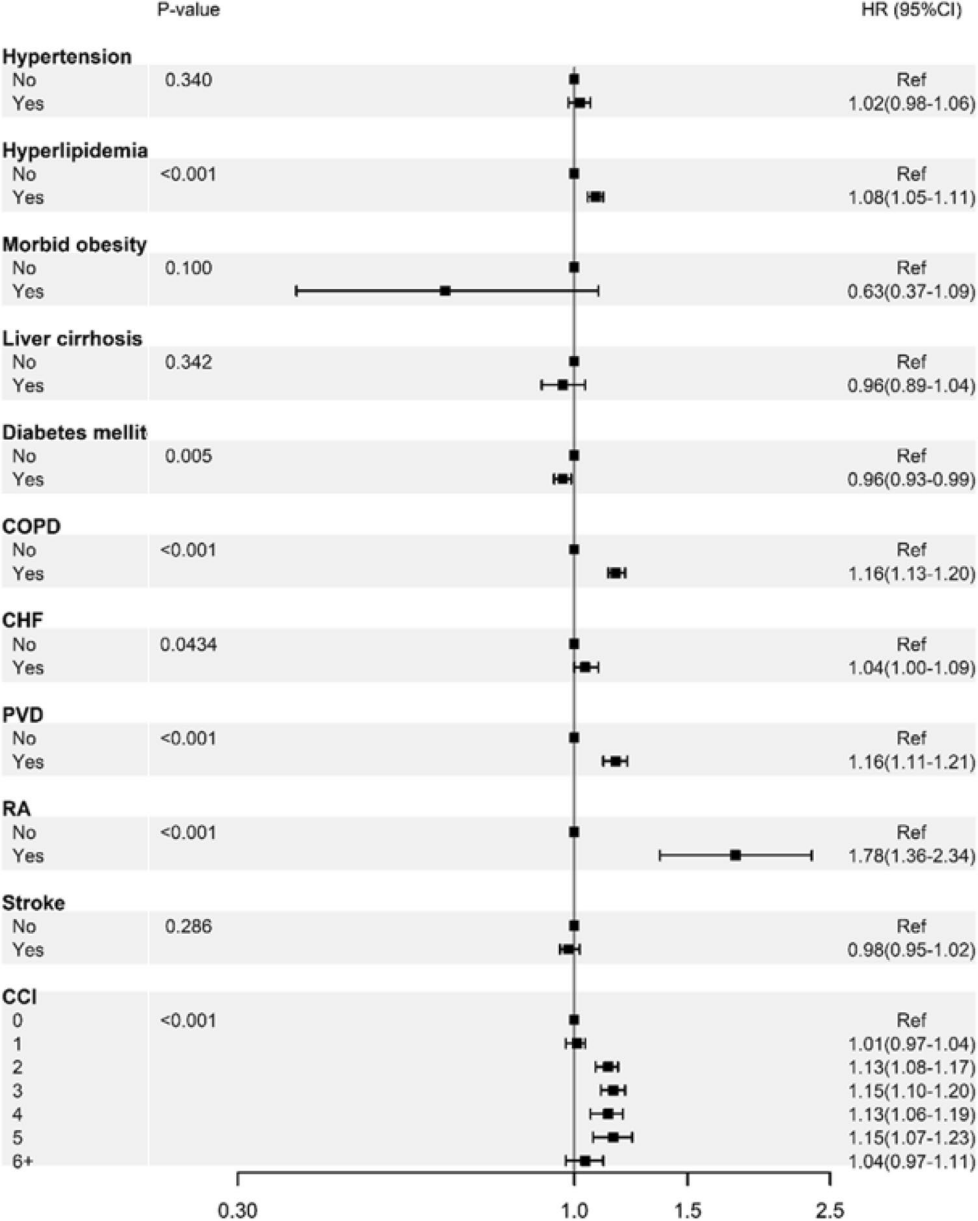
Cox model–derived crude and multivariate adjusted hazard ratios (HRs) of osteoporosis development stratified by patient characteristics

Figure 2 shows the multivariate Cox model analysis of the differential risk among different categorizations, such as hyperlipidemia, for patient characteristics, with the adjusted HR of OP stratified by each patient characteristic. The patients with hyperlipidemia had an increased risk of OP (adjusted HR 1.08 95% CI, 1.05–1.11), as shown in Fig. 2. Moreover, patients with COPD, PVD, and RA had an increased risk of OP compared with those who did not have these comorbidities (Fig. 2). Additionally, patients with a CCI score of 2–5 also had a significantly increased risk of OP compared with patients who had a CCI score of 0.

**Fig. 3:**
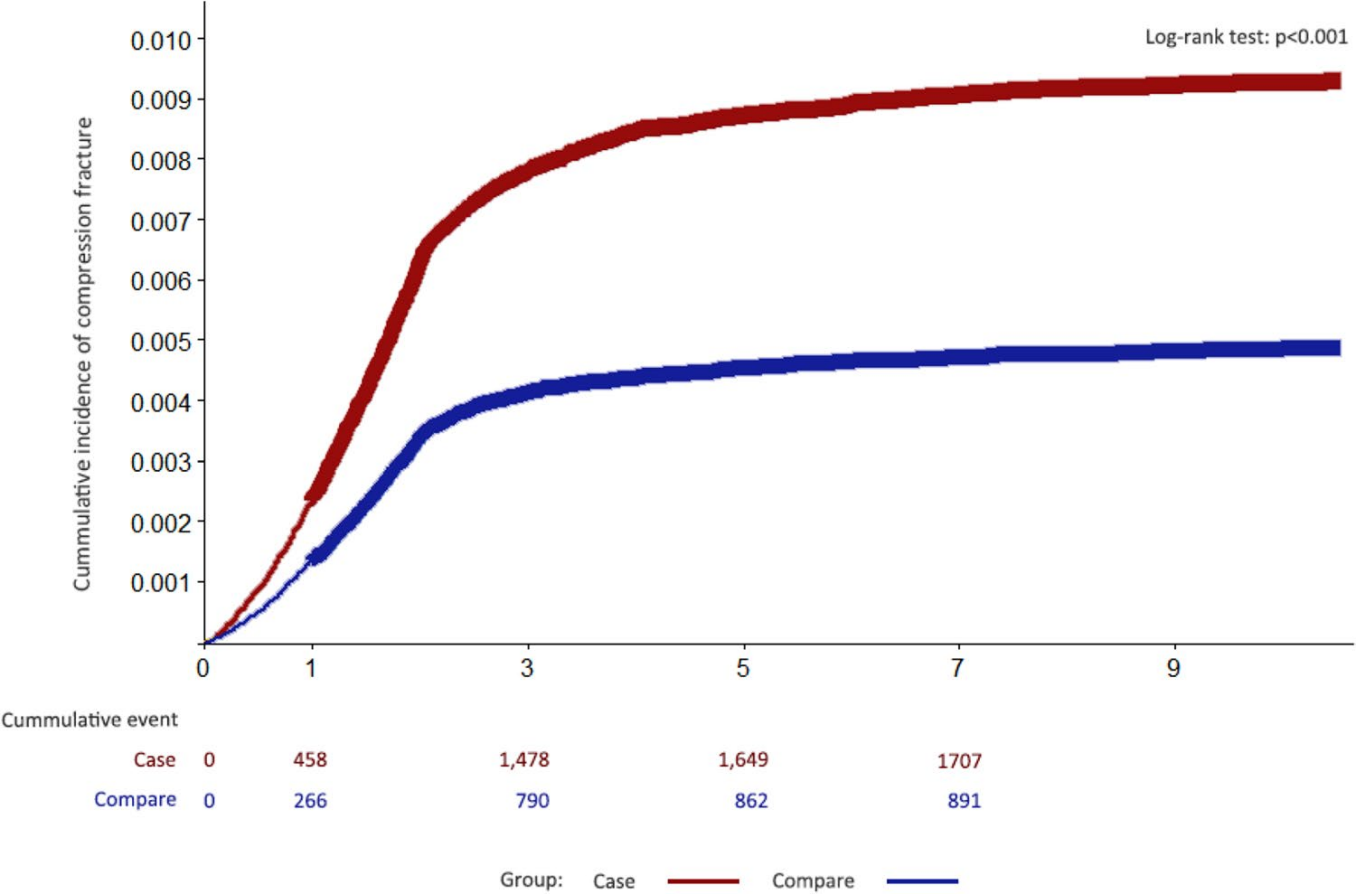
Cumulative incidence of thoracolumbar vertebral compression fracture, which is 0.9% and 0.47%, respectively, in the CAD and non-CAD cohorts (*p* < 0.0001, compared with log-rank)

**Fig. 4:**
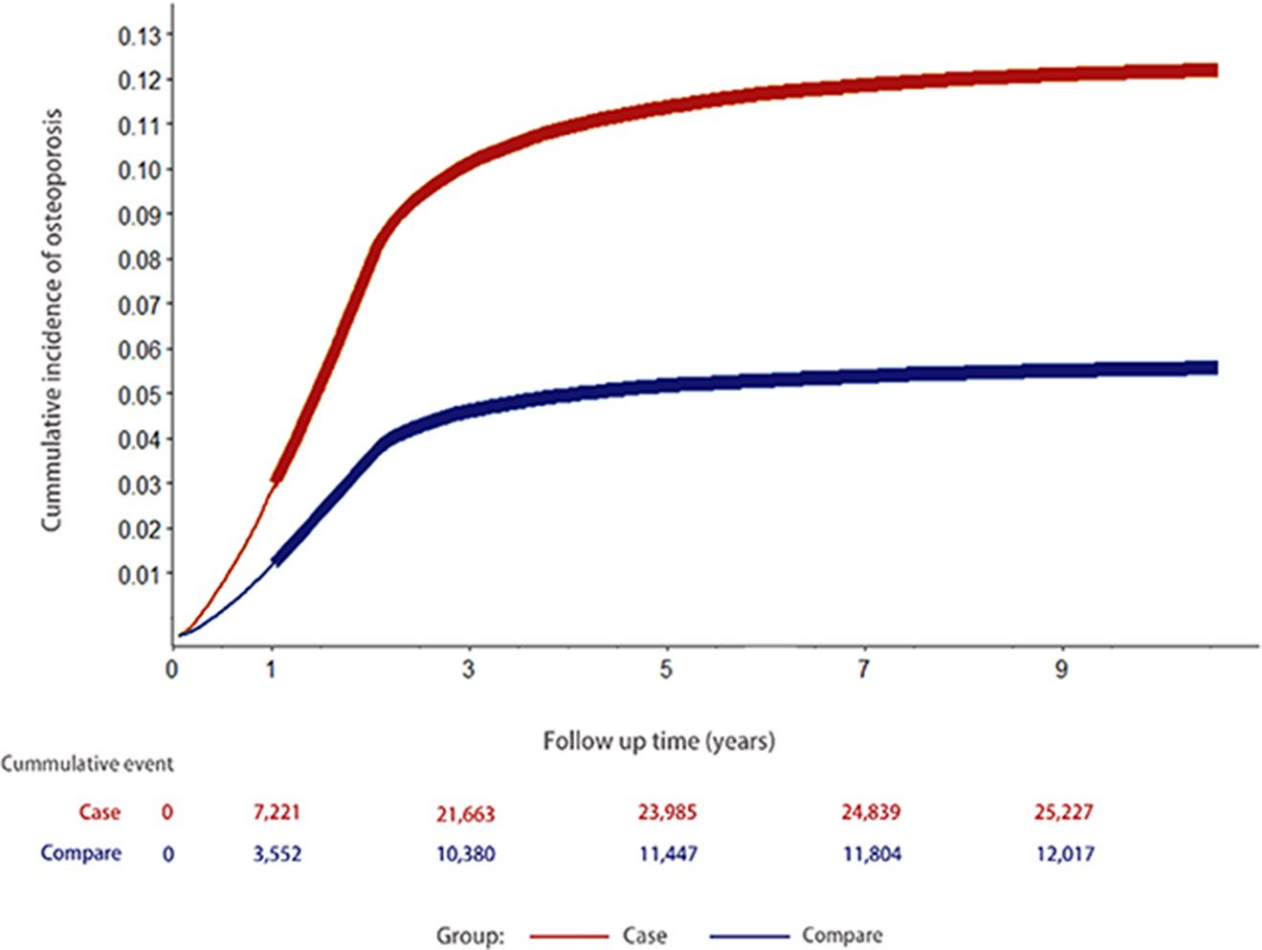
Cumulative incidence of osteoporosis, which is 11.6% and 5.8%, respectively, in the CAD and non-CAD cohorts (*p* < 0.0001, compared with log-rank)

We constructed a multivariate Cox model to derive the cumulative incidence function excluding the first year of follow-up events. The cumulative incidence of osteoporotic fracture is 0.9% and 0.47% (*p* < 0.001, log-rank) for the CAD and non-CAD cohorts (Fig. 3). For the secondary outcome, Fig. 4 shows the cumulative incidence of OP for the CAD and non-CAD cohorts, which is 11.6% and 5.6%, respectively (*p* < 0.001, log-rank).

## Discussion

The understanding of the pathophysiologic factors for and associations shared by CAD and OP has increased over the recent past years. Unfortunately, no consistent results have been shown to date, and both diseases have yet to be investigated adequately with regard to the etiologic role of CAD as a possible risk factor for OP. To our knowledge, this study is the first to examine data from the NHIRD as a national population-based study to analyze the association between OP and CAD in Taiwan. Our study discovered a significant increase in the risk of developing a vertebral compression fracture for patients with CAD compared with their non-CAD counterparts based on the multivariate Cox model (adjusted HR 1.74). Additionally, the CAD group also had a higher incident rate of osteoporosis than the control group. Overall, a positive association between CAD and subsequent OP development was shown in this study.

Previous studies have demonstrated that age-related bone loss and cardiovascular disease may be related to chronic inflammation [20, 21]. Elevation of osteoprotegerin, sclerostin, or FGF-23 levels may explain and predict the occurrence of both OP fractures and cardiovascular events [13, 22, 23]. On the basis of these pathophysiologic studies, there is an association between the OP and CAD. Our study also provided evidence of an association between the two diseases. However, to clarify the mechanisms for and the association between CAD and OP, a more well-designed study is needed to establish stronger data for the pathophysiologic association.

Two recent meta-analyses have revealed controversial results for the association between OP and CAD. One meta-analysis assessed 4170 participants from 11 studies. The pooled odds ratio (OR) for the incidence of CAD in patients with low BMD versus patients with normal BMD was 1.58 (95% CI, 0.99–2.52; *p* = 0.06), and no statistical difference was found between the subgroups of men and women. After analyzing the confounding age, the combined OR was 1.60 (95% CI, 0.69–3.72; *p* = 0.27) [16]. Therefore, their conclusion was that the low BMD was not associated with the prevalence of CAD. The other meta-analysis assessed 10,299 patients from 25 studies. They found that the incidence of atherosclerotic vascular abnormalities was significantly increased in patients with low BMD, compared with patients with normal BMD (OR 1.81; 95% CI, 1.01–2.19; *p* < 0.00001). After the study adjusted for age, sex, body mass index (BMI), and other vascular risk factors, decreased BMD remained significantly associated with the incidence of atherosclerotic vascular abnormalities (OR 2.96; 95% CI, 2.25–3.88; *p* < 0.00001) [24]. These findings showed that decreased BMD was an independent predictor for the development of atherosclerosis in older adults.

Some cohort studies evaluated whether lower bone mass affects cardiovascular events. One cohort study consisted of 5590 consecutive at-risk patients without known CAD, age 57 ± 12 years, and 69% male, who underwent nonenhanced cardiac computed tomography, followed for an average of 8 years. Analysis of the clinical outcomes of CAC scanning and thoracic BMD measurement showed a significant link between low BMD levels and CAC with an increased risk of mortality in both sexes across ethnicities. They claimed that the lower BMD levels are independently associated with the severity of CAC that predicts mortality [15]. Another prospective cohort study in France included 744 men older than 50 years with a 7.5-year follow-up. They found 40 patients had several myocardial infarction, and 43 patients had a stroke [25]. After adjusting for risk factors, the patients whose BMD was in the lowest quartile or whose bone resorption markers were in the highest quartile had a twofold higher risk of cardiovascular events. Furthermore, after adjustment, BMD was significantly lower in the patients with stroke. Last, a prospective cohort study in Sweden evaluated 6872 men and women for 5.7 years, during which time 196 patients experienced myocardial infarction [26]. Myocardial infarction was then concluded to be significantly associated with low hip BMD (women, OR 1.33; 95% CI, 1.08–1.66; men, OR 1.74; 95% CI, 1.34–2.28).

Our current study had several strengths, including the large sample size, randomization, and sufficient follow-up duration. The data originated from a national population dataset, which provided the appropriate statistical power for detecting the association between the two diseases. Additionally, when analyzing a random matching of the non-CAD cohort, it was well balanced for the index date, sex, and age. However, this study has several limitations. First, possible coding errors and other coding problems may occur in the administrative databases. For our study, the definition of CAD was based on the coding in the database, but the accuracy between the diagnosis and coding was not verified. To minimize the interference, we added the restriction that the CAD diagnosis must exist at or more two ambulatory care visits or at one or more inpatient visits and must be made by certain specialists. Second, data were limited or unavailable for serum 25-hydroxyvitamin D; serum parathyroid hormone; proinflammatory cytokines such as IL-1, IL-6, and TNF-α; and serial BMD values, so these factors could not be analyzed further. Additionally, BMI and lifestyle, such as individual dietary habits and physical activity, data related to CAD and OP were not recorded in the dataset. Because this study showed a positive association between CAD and OP, it is worthy of initiating well-designed in vivo or in vitro studies to investigate causation and pathophysiologic link between the two diseases in the future.

## Conclusions

In conclusion, patients with CAD have a significant increase in the risk of developing vertebral compression fracture and osteoporosis than those without CAD. This study has demonstrated the positive association between CAD and subsequent development of vertebral compression fracture and OP. To prevent the complications of OP, patients with CAD should receive a personalized risk assessment and screening for OP.

## Data Availability

These data were available to us as staffs of the Department of Orthopedics at Fu Jen Catholic University Hospital and at Fu Jen Catholic University, using the National Hospital Research Database (NHIRD). These data are protected by the Ministry of Health and Welfare and patient privacy laws in Taiwan; no public links are available to these protected health information datasets. These data will be made available to others after appropriate data privacy and human subject approvals needed by the institution. Requests should be sent to 081438@mail.fju.edu.tw.
